# New implications from long-term outcomes of perioperative therapy in resectable pancreatic cancer

**DOI:** 10.1038/s41416-025-03295-9

**Published:** 2025-12-04

**Authors:** Christoph Springfeld, Thilo Hackert, Daniel H. Palmer, Daniel Öhlund, Teresa Peccerella, Thomas Hank, Markus W. Büchler, Christoph W. Michalski, John P. Neoptolemos

**Affiliations:** 1 Department of Medical Oncology, National Center for Tumor Diseases, Heidelberg University Hospital, Heidelberg, Germany; 2 Department of General, Visceral and Thoracic Surgery, University Hospital Hamburg-Eppendorf, Hamburg, Germany; 3 Cancer Research UK Liverpool Experimental Cancer Medicine Centre, University of Liverpool, Liverpool, UK; 4 Department of Diagnostics and Intervention (Oncology), and Wallenberg Centre for Molecular Medicine (WCMM), Umeå University, Umeå, Sweden; 5 Department of General, Visceral and Transplantation Surgery, Heidelberg University Hospital, Heidelberg, Germany; 6 Champalimaud Foundation, Lisbon, Portugal

**Keywords:** Pancreatic cancer, Surgical oncology

## Abstract

The biggest impact on increasing survival for pancreatic cancer has come about by combining surgical resection with systemic chemotherapy. This groundbreaking paradigm has come under increasing scrutiny relating to the choice of adding chemoradiotherapy to chemotherapy versus chemotherapy alone, neoadjuvant versus adjuvant therapy and the optimal regimens. The paradigm has also been challenged in that a distinction needs to be made between ‘resected’ with ‘resectable’ pancreatic cancer, since if only the former is considered, this leads to a biased prognostically favourable patient group being analysed. Moreover, the distinction between resectable, borderline resectable and unresectable cancers is claimed to be so unreliable that this classification should be discouraged in favour of upfront chemotherapy for all patients and not necessarily using either FOLFIRINOX or gemcitabine-capecitabine. The results of a series of recent trials including the RTOG0848 trial of adjuvant chemotherapy with or without chemoradiation and the NORPACT-1 trial of neoadjuvant FOLFIRINOX versus upfront surgery for resectable pancreatic cancer have significantly contributed to the clarification of some these questions. The results of long-term follow-up studies of the adjuvant PRODIGE24 trial comparing FOLFIRINOX with gemcitabine and the ESPAC4 trial of gemcitabine-capecitabine versus gemcitabine have also consolidated and expanded the applicability of adjuvant chemotherapy.

## Adjuvant chemotherapy for resectable pancreatic cancer challenged as the established reference standard

The pivotal European Study Group (ESPAC) ESPAC1 and ESPAC1-Plus trials published in the Lancet and New England Journal of Medicine in 2001-2004, showed that post-operative adjuvant chemotherapy but not radiochemotherapy substantially improved overall and 5 year survival rates compared to surgical removal alone in patients with resectable pancreatic ductal adenocarcinoma (PDAC) [1, 2]. These trials were based on using only 5-fluorouracil (5FU) monotherapy (with the 5FU enhancer leucovorin) as the adjuvant therapy, and received considerable scepticism and criticism especially from radiation oncologists in the USA [3, 4]. Further studies from the ESPAC, Charité Onkologie (CONKO) and Japan Adjuvant Study Group of Pancreatic Cancer (JASPAC) teams have confirmed the survival advantage of adjuvant monochemotherapy not only with 5FU but also with gemcitabine, and the oral agent S1 (comprising the 5FU prodrug tegafur, plus the enhancers gimeracil and oteracil potassium) [5–8].

These trials evolved into adjuvant combination chemotherapy regimens providing even greater overall and 3–5 year survival rates [9, 10]. The ESPAC4 study showed improved overall survival with the combination of gemcitabine with the oral 5-FU prodrug capecitabine (GemCap) finding an increased 5-year overall survival of 28.8% compared to 16.3% for gemcitabine monotherapy [9]. The ESPAC4 study had relatively unrestricted eligibility criteria with no upper age limit and no constraints on serum CA19-9 levels [9]. The France-Canada PRODIGE24-CCTG PA.6 trial demonstrated even more striking results using modified (m)FOLFIRINOX (comprising 5-FU, FA, irinotecan and oxaliplatin) with a 3-year overall survival of 63.4% compared to 48.6% using gemcitabine [10]. mFOLFIRINOX however was associated with greater toxicity than gemcitabine and the PRODIGE24 trial was more stringent in its selection criteria, requiring patients aged 79 years or less, with a WHO performance status of 0 or 1, no significant cardiovascular disease and a postoperative serum tumour marker CA19-9 level <180 KU/L [10]. In real world practice, this additional toxicity burden translates into less than 50% of patients receiving adjuvant chemotherapy having mFOLFIRINOX, falling to less than 10% of those aged >70 years [11, 12].

Interestingly, the APACT study comparing gemcitabine plus nab-paclitaxel against gemcitabine did not meet its primary end point of independently assessed median disease free survival (19.4 versus 18.8 months respectively, hazard ratio [HR] of 0.88 with 95% confidence [CI], 0.729–1.063; *P* = 0.18), although the median investigator-assessed disease free survival was significant (16.6 versus 13.7 months respectively, HR of 0.82 [95% CI, 0.694–0.965]; *P* = 0.02), and also overall survival was not significantly different initially but was on further follow up [13]. This apparent discrepancy highlights the challenges of surrogate endpoints such as disease free survival and local investigator versus independent review for adjuvant studies in PDAC. At the 5-year follow-up point the median overall survival was 41.8 months for gemcitabine plus nab-paclitaxel versus 37.7 months for gemcitabine monotherapy, with 5 year overall survival rates of 38% and 31% respectively with an HR of 0.80 (95% CI, 0.678–0.947; *P* = 0.0091) [13]. Gemcitabine plus nab-paclitaxel is not approved by the FDA as adjuvant therapy for pancreatic cancer. The current European Society of Medical Oncology (ESMO) pancreatic cancer guidelines also state that there is no role for gemcitabine plus nab-paclitaxel in the adjuvant setting, whilst mFOLFIRINOX is the established reference standard for fit patients, with GemCap as an option, although typically this combination is now reserved for patients not eligible for, or choosing not to have, mFOLFIRINOX [14].

A post hoc analysis of the ESPAC3 trial identified completion of all six cycles of adjuvant chemotherapy as a key favourable prognostic factor for overall survival rather than starting the chemotherapy earlier after resection, which was later confirmed by the PRODIGE24/CCTG PA.6 trial [15, 16].

The CONKO-001 trial when first reported only found a significant difference for disease free survival with gemcitabine compared to observation but with prolonged follow up there was significant overall survival difference (Table 1) [5, 17]. Extended median follow up of 69.7 months in the PRODIGE24/CCTG PA.6 trial showed sustained improvement in median survival differences and a 5 year overall survival rate 43.2% for mFOLFIRINOX versus 31.4% for gemcitabine [16]. With further follow up now of 104 months in the ESPAC4 trial there was a sustained median overall survival of 31.6 months in the GemCap group compared to 28.4 months with gemcitabine alone, translating into 5 year survival estimates of 32% (95% CI, 27–36) and 25% (95% CI, 21–30), respectively [18].

**Table 1:** Anatomical (empirical) surgical staging for non-metastatic pancreatic cancer.

Resection Stage	Staging System	
	Alliance for Clinical Trials in Oncology—Alliance [31]	National Comprehensive Cancer Network—NCCN [19]
Superior Mesenteric Vein-Portal Vein		
Resectable	Interface between tumour and vessel measuring <180°	No tumour contact or ≤180° contact without vein contour irregularity
Borderline Resectable	Interface between tumour and vessel measuring ≥ 180°, and/or reconstructable^a^ occlusion	Solid tumour contact measuring >180°, or solid tumour contact ≤180° with contour irregularity or thrombosis
Locally Advanced	Unreconstructable	Unreconstructable
Superior Mesenteric Artery		
Resectable	No interface between tumour and vessel	No solid tumour contact
Borderline Resectable	Interface between tumour and vessel measuring < 180°	Solid tumour contact ≤180°
Locally Advanced	Interface between tumour and vessel measuring ≥ 180°	Solid tumour contact >180°
Common Hepatic Artery or its First Order Branches		
Resectable	No interface between tumour and vessel	No solid tumour contact
Borderline Resectable	Reconstructible^a^, short-segment interface between tumour and vessel of any degree	Solid tumour contact without extension to coeliac or hepatic artery bifurcation
Locally Advanced	Unreconstructable	Unreconstructable
Coeliac Trunk		
Resectable	No interface between tumour and vessel	No solid tumour contact
Borderline Resectable	Interface between tumour and vessel measuring < 180°	Solid tumour contact ≤180°
Locally Advanced	Interface between tumour and vessel measuring ≥ 180°	Solid tumour contact >180°

^a^Normal vein or artery proximal and distal to the site of suggested tumour-vessel involvement suitable for vascular reconstruction.

Despite the ESMO recommendations being consistent with other international guidelines notably the USA National Comprehensive Cancer Network (NCCN) Clinical Practice Guidelines in Oncology and the England National Institute for Clinical Excellence (NICE) in recommending adjuvant chemotherapy as standard practice for resectable PDAC, there are a number of challenges to this position [19, 20]. Firstly, it is argued that that a distinction needs to be made in clinical trials comparing ‘resected’ with ‘resectable’ pancreatic cancer, since if we only consider the former then the inclusion criteria are biased leading to a prognostically false favourable patient group being analysed [21, 22]. Secondly the distinction between ‘resectable’ and ‘borderline resectable’ groups is so unreliable these should be considered as single entity that we refer to as ‘resectable(*)’ [23–25]. Reni and Orsi stated ‘that surgical classification (i.e. distinguishing between resectable, borderline resectable and unresectable pancreatic adenocarcinomas), apart from lacking prognostic validation, is irreproducible and its use should be discouraged’ [26]. Thus we can only consider two categories of clinically relevant patient groups namely non-metastatic (1) locally ‘resectable (*)’ and (2) ‘unresectable’. In which case and thirdly all ‘resectable(*)’ and ‘non-resectable’ patients should be treated in the same way with upfront systemic therapies, using neoadjuvant and induction combination chemotherapy regimens [27]. Finally according to Reni and colleagues since mFOLFIRINOX and GemCap have never been tested in the ‘resectable (*)’ setting then neither can be considered the reference standard, and they recommend consideration of alternative regimens such as PAXG (cisplatin, nab-paclitaxel, capecitabine and gemcitabine) which in the case of PAXG has been tested in ‘resectable(*)’ patients [28]. Further analysis of these arguments using published data also needs to be based on a thorough understanding of modern surgical developments as the cornerstone of multi-modality treatments.

## Advances in surgery underpinning multi-modality treatment

**Fig. 1:**
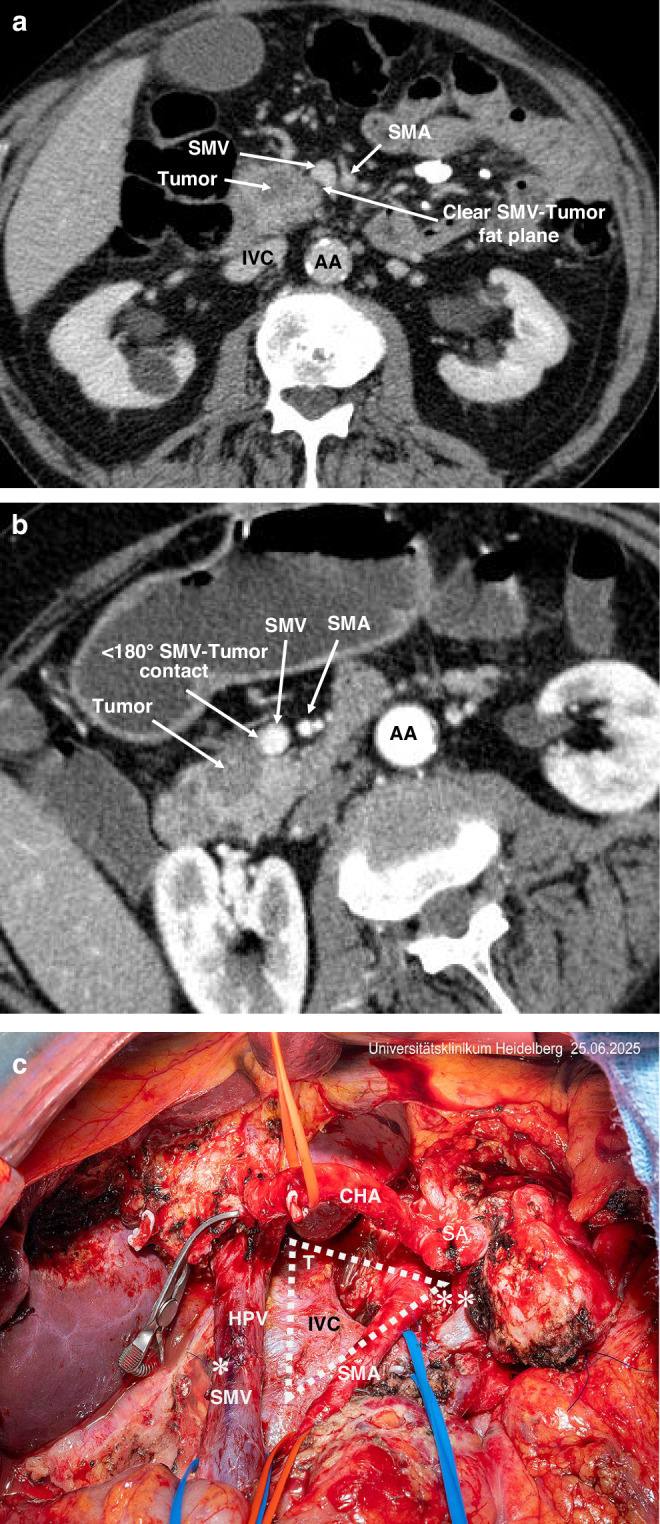
Preoperative co-axial imaging by CT demonstrating the distinction between resectbale (EmR) and borderline (EmBR) PDAC, and the key features of the TRIANGLE operation. **a** CT image of resectable cancer in the head of the pancreas. **b** CT image of borderline resectable cancer in the head of the pancreas. **c** Surgical site after partial pancreato-duodenectomy with venous resection, triangle dissection and splenorenal shunt for borderline rectable disease following induction chemotherapy disease. AA abdominal aorta, CHA common hepatic artery, SMA superior mesenteric artery, HPV hepatic portal vein, SMV superior mesenteric vein, SA splenic artery, IVC inferior vena cava, * hepatic portal vein resection, ** splenorenal shunt, T triangle.

Whilst surgery remains the only curative modality for a small proportion of patients with non-metastatic PDAC, its role and scope have significantly evolved in the era of multimodal therapy with very low post-operative mortality rates and improving long-term survival and good quality of life measures [29, 30]. Anatomical/empirical criteria for resectability, internationally accepted and applied in the form of the Alliance and NCCN staging systems widely guide surgical decisions (Table 1; Fig. 1a, b) [19, 31]. There is also increasing recognition of the desirability of considering biological parameters in the classification of resectability [32, 33]. At the present time however attempted staging by tumour biology is rather crude, using parameters such as CA19-9 levels and lymph node involvement [32, 33]. Given the evolutionary clonality of PDAC accurate tumour biologic staging requires integrating the genotypic (DNA) and phenotypic (mRNA) features of the PDAC tumour necessitating biopsies for sequencing and assessment of tumour microenvironment subtypes [34–36]. Liquid biopsies for tumour biotyping and minimal residual disease detection are still at a relatively early stage of evolution but will be of increasing importance with technology advancements [37, 38]. Radical surgery including adequate lymphadenectomy, vascular resection and retroperitoneal clearance is essential to achieving margin-negative resections (R0) and optimising long-term outcomes in patients with empirical classification (Em) resectable (EmR) and borderline resectable (EmBR) disease [39, 40].

In patients with EmR, the tumour is anatomically resectable, CA19-9 is within an acceptable range and the patient’s performance status supports upfront surgery followed by adjuvant chemotherapy remains the standard of care [30, 41–43]. The precise CA19-9 level favouring surgery is not fully established, although a post-operative upper limit of normal of ≤2.5 as in CONKO-01 or <180 KU/L as in PRODIGE24 are often cited, and according to ESMO guidelines a preoperative serum CA19-9 level ≥500 KU/L indicates a worse prognosis after surgery and immediate surgery should be considered with caution [10, 14, 17, 19]. The operative approach for EmR tumours typically includes partial pancreato-duodenectomy, left (distal) pancreatectomy with splenectomy, or total pancreatectomy, depending on the exact tumour location [44]. In tumours with contact to the coeliac axis or the superior mesenteric artery (SMA), an artery-first approach, via posterior, medial, or inferior approaches, is performed to reduce blood loss, enable early assessment of resectability and to avoid R2 resections [45]. A systematic lymphadenectomy is performed, including complete clearance of lymphatic tissue along the right aspect of the SMA and the coeliac trunk [39, 46]. By achieving an R0 resection in the upfront setting a median overall survival is achieved of 41.6 months with a 5-year survival rate of 37.7% for pancreatic head tumours which is even higher for pancreatic body and tail tumours with 62.4 months and a 5-year survival rate of 52%, respectively [40, 47, 48].

Patients with EmBR tumours often present with anatomical findings such as venous abutment, short-segment involvement of the portal vein or SMV, compression of the SMA, and elevated CA19-9 [49, 50]. Though technically resectable, these tumours are associated with higher risk of R1 resection and early relapse. Neoadjuvant chemotherapy increases survival in this setting irrespective of resectability although surgical resection remains the central potentially curative step in this setting [30, 34]. Surgical resection in EmBR disease frequently involves venous resection and reconstruction and in selected cases, arterial divestment or resection [49, 50]. Besides this the TRIANGLE dissection, involving en bloc skeletonization of the SMA, celiac axis and portal vein has emerged as the benchmark approach for tumours located in the pancreatic head, allowing clearance of perineural and lymphovascular tissue planes commonly harbouring residual disease (Fig. 1c) [51]. To gain high-level evidence for this approach a randomised controlled trial is now ongoing in Germany [52].

Surgical management of resectable and borderline resectable PDAC must be performed in centres with high case volume, multidisciplinary decision-making and excellent perioperative care. Mortality rates for PDAC surgery in such centres have fallen below 5%, and morbidity, although still significant, can be mitigated through standardised pathways (e.g. drain management, early sepsis detection, enhanced recovery). Failure to rescue, defined as mortality after a major complication, is now recognised as a text book outcome benchmark of surgical quality [53]. While more radical surgery including vascular resection and extended dissection may carry increased risk, evidence suggests that when performed in appropriate centres, these techniques do not increase mortality and can significantly improve long-term survival by achieving margin-negative resection in biologically fit patients.

## Adjuvant chemoradiotherapy controversy

Whilst the role of adjuvant chemotherapy has been consolidated these past 25 years through well designed randomised trials, the controversy over the role of chemoradiation has never quite settled amongst radiation oncologists especially in the USA (Table 2) [3, 4, 54–65]. The Radiation oncology Group (RTOG) led by Ross Abrams responded to the ESPAC gamechanger by designing a 2-step adjuvant chemotherapy and chemoradiation trial [61, 62]. This was essentially with a 2 × 2 factorial design that had previously been heavily criticised in ESPAC1 [3, 4]. The RTOG0848 trial initially (step 1) randomised patients to 5 cycles of gemcitabine with or without erlotinib, and if there was no disease relapse patients were then further randomised (step 2) to either a further cycle of gemcitabine or combination chemotherapy or to a further cycle of gemcitabine or combination chemotherapy plus chemoradiation with either capecitabine or 5FU based chemoradiation [61, 62]. Recruitment commenced in 2009, the results of patients randomised in step 1 were published in 2020, and the final results of patients randomised to step 2 were recently reported following complete target accrual in October 2018 (Table 1) [61, 62]. Despite the headline finding that the trial failed to achieve its primary endpoint of improved overall survival with the addition of chemoradiotherapy, it is still being promoted for patients with negative lymph nodes [62]. This small subgroup had a median overall survival of 3.9 years in the chemoradiation group (*n* = 49) compared to 3.0 years in the chemotherapy group (*n* = 42), with 5-year overall survival estimates of 48.1% and 28.6% respectively [62]. It is noteworthy that there was no survival benefit for chemoradiation for the patients with positive margins (R1) or positive lymph nodes, yet these are the same exact subgroups that had been advocated for previously as benefitting from chemoradiotherapy [62]. Methodologically it would be difficult to support this specific use given the post hoc nature of the analysis and the prior unspecified application of a one-sided alpha that was used to show significance (*p* = 0.03) [62]. Contrasting these findings, the ESPAC4 study found that the 5-year overall survival rate for node-negative patients was 59% for patients receiving GemCap and 53% for patients just receiving gemcitabine [18]. The ESMO pancreatic cancer guidelines do not recommend adjuvant chemoradiotherapy and it should not be given to patients following surgery outside the setting of a clinical trial [14]. Understanding the biological basis of the failure of chemoradiotherapy to impact on any improvement in survival in pancreatic cancer is essential in order to determine whether this modality can be used in some way in pancreatic cancer compared to other potentially effective therapies [66–69].

**Table 2:** Randomised adjuvant trials in resectable pancreatic cancer since publication of the ESPAC1 and ESPAC1-Plus studies.

Trial	Recruitment period	Treatment arms	Number of patients	Median overall survival (months)	5-year overall survival (%)	Comments
**ESPAC1Plus** [1] **All patients and early follow-up of 2 × 2 factorial patients**	1994–2000	No CRT	178	16.1	19.5	ECOG 0,1,2; R0/R1. Significant for chemotherapy overall but not in the 2 × 2 factorial. Not significant for CRT overall or in 2 × 2 factorial.
		CRT	175	15.5 *P* = 0.24	10.3	
		No chemotherapy	235	14.0	9.9	
		5FU/FA	238	19.7 *P* = 0.0005	23.3	
**ESPAC1** [2] **2 × 2 factorial, final follow up**	1994–2000	No CRT	144	17.9	19.6	ECOG 0,1,2.
		CRT	145	15.9 *P* = 0.05	10.8	R0/R1.
		No chemotherapy	142	15.5	8.4	
		5FU/FA	147	20.1 *P* = 0.009	21.1	
		Observation	69	16.9	10.7	
		CRT	73	13.9	7.3	
		5FU/FA	75	21.6	29.0	
		CRT + 5FU/FA	72	19.9	13.2	
**CONKO-001** [5, 17]	1998–2004	GEM	179	22.8	20.7	Post-operative CA19-9 > 92.5
		Observation	175	20.2 *P* = 0.01	10.4	KU/L = 0.0%.
**EORTC 40891** [54]	1987–1995	CRT	60	24.5	20	T1-2, N0-1a, M0 pancreatic head cancer.
		Observation	54	19 (*P* = 0.099)	10	
**RTOG 9704** [55, 56]	1998–2002	5FU/FA, 5FU + RT, 5FU/FA	230	–	–	Median survival only reported in 388 with pancreatic head tumours = 20.5 (GEM) vs. 16.9 (5FU) months, *P* = 0.09.
		GEM, 5FU-RT, GEM	221	- *P* = 0.34	–	
**ESPAC-3** [7]	2000–2007	5FU/FA	551	23.0	15.9	ECOG 0,1,2; R0/R1.
		GEM	537	23.6 *P* = 0.39	17.5	
**JSAP-02** [57]	2002–2005	GEM + IORT in 27	58	22.3	23.9	Karnofsky > 50.
		IORT in 47 then observation	60	18.4 *P* = 0.19	10.6	
**CapRI** [58]	2004–2007	5FU, cisplatin, IFNα2b + RT, CI 5FU	64	32.1	25	ECOG 0,1,2; R0/R1.
		5FU/FA	68	25.5 *P* = 0.49	25	
**JASPAC-01** [8]	2007–2010	GEM	190	25.2	24.4	ECOG 0 = 68.7%; post-op. CA19-9 > 37 KU/L = 21%; R1 pos. = 31%; LN pos. = 62.9%.
		S-1	187	46.5 *P* < 0.0001	44.1	
**CONKO-005** [59]	2008–2013	GEM	217	26.2	20.0	Karnofsky PS ≥ 60%
		GEM-erlotinib	219	24.5 *P* = 0.061	25.0	Only R0 resected patients.
**CONKO-006** [60]	2008–2013	GEM	65	17.1	–	Karnofsky PS ≥ 60%.
		GEM-sorafenib	57	18.2 *P* = 0.94	–	Only R1 patients.
**ESPAC-4** [9, 18]	2008–2014	GEM	366	28.4	25.0	R0 and R1 patients.
		GEMCAP	365	31.6 *P* = 0.031	32.0	
**NRG Oncology/RTOG 0848** [61, 62] 2-step trial	2009–2014	GEM	163	29.9	–	Post-op. CA 19-9 < 180 KU/L.
		GEM + erlotinib	159	28.1 *P* = 0.62.	–	Step 1: 5 cycles GEM +/-erlotinib. Step 2: 6th cycle GEM+/-CRT.
		Chemotherapy	174	31.0	23.0	1-sided test.
		Chemotherapy +CRT	180	27.0 *P* = 0.38	28.0	
**PRODIGE-24** [10, 16]	2012–2016	GEM	246	35.5	31.4	ECOG 0,1. Post-op CA 19-9 < 180 KU/L. <80 years.
		mFOLFIRINOX	247	53.5 *P* = 0.001	43.2	
**APACT** [13]	2014–2018	GEM	434	37.7	31.0	ECOG 0,1; post-op CA 19-9 < 100 KU/L; primary endpoint DFS, not met. 18.0 (GEM) vs 19.4 (GEM + NabP) months, *P* = 0.1824.
		GEM-NabP	432	41.8 *P* = 0.009, Not primary end point	38.0	

*CRT* chemoradiotherapy, *RT* radiotherapy, *CTX* chemotherapy, *CI* continuous infusion, *5FU* 5-fluorouracil, *DOX* doxorubicin, *MMC* mitomycin C, *FA* folinic acid, *GEM* gemcitabine, *CAP* capecitabine, *IORT* intra-operative radiotherapy, *mFOLFIRINOX* modified folinic acid (FA), 5-fluorouracil (5FU), irinotecan (IR) and oxaliplatin (OX), *NabP* nab-paclitaxel, *DFS* disease free survival, *OS* overall survival.

## Neoadjuvant therapy is not superior to adjuvant chemotherapy

Dissipation of the notion that adjuvant chemoradiotherapy was a ‘must have’ in the treatment of pancreatic cancer slowly followed solidification of evidence against its use from 2000 onwards. Pari passu was the emergence of a similar notion that neoadjuvant therapy (including chemoradiotherapy) is a ‘must have’ for all stages of pancreas cancer including resectable pancreatic cancer [70, 71]. This opinionated perception fails to take into account the most modern concept of tumour plasticity which reflects the continuing biological evolution of PDAC, determined by temporal and intrinsic and extrinsic tumour microenvironment pressures, that are quite independent of standard TNM UICC and AJCC classifications [68, 72–75]. This concept translates into a new understanding that not all pancreatic cancers are the same but are biologically different even though they may appear to be similar macroscopically and histologically. The empirical classification (Em) of resectable (EmR), borderline resectable (EmBR), locally advanced unresectable (EmUR), oligometastatic (EmOM), and polymetastatic (EmPM), equates to a distinct biological transitioning of stages, that requires differing treatment strategies [34, 76]. Thus, an effective therapeutic sequence strategy for one empirical stage cannot automatically be assumed to be applicable to another [32, 74].

Randomised trials demonstrate that whilst neoadjuvant approaches have shown improvement in overall survival in patients with EmBR tumours this has not been the case for patients with EmR tumours (Table 3) [28, 77–89]. A number of studies (notably PREOPANC1, PREOPANC2, and most recently Prep-02/JSAP05 and CASSANDRA) have caused considerable confusion in interpretation by combining recruitment of patients with both EmR and EmBR tumours and drawing conclusions for both when this might not be the case as in PREOPANC1which showed a neoadjuvant therapy survival benefit in the patients with EmBR tumours but not those with EmR [28, 80, 81, 86, 88]. The Prep-02/JSAP05 trial is a case in point, having originally presented the results at the Asssociation of Clinical Oncolgy Meeting in 2019, the full manuscript has only just now been published [88]. This multicenter trial from Japan recruited 364 patients that were randomly allocated to upfront surgery (*n* = 182) and were ‘*strongly advised*’ to take adjuvant S1, or neoadjuvant gemcitabine plus S-1 followed by surgery and also ‘*strongly advised*’ to take adjuvant S1 (*n* = 182) [88]. The distinction between EmR and EmBR was ambiguous throughout the manuscript. The primary endpoint itself was ‘to confirm the superiority of neoadjuvant therapy with GS (gemcitabine plus S-1) over the standard strategy of upfront surgery in patients with resectable PDAC.’ Enrolment took place between January 2013 and January 2016, and analysis was undertaken using data collected up to September 2018, 2 years and 9 months after the final enrolment. The median overall survival was 26.6 months (95% CI, 21.5–31.5) in the upfront surgery group and 37.0 months (95% CI, 28.6–43.3) in the neoadjuvant group (*p* = 0.018) [88]. Critically, the protocol is not available and there is no mention of the statistical analysis plan.

**Table 3:** Randomised trials of neoadjuvant treatment for resectable pancreatic cancer.

Trial	Recruitment period	Treatment arms	Number of patients	Median overall survival (months)	Comments
Germany multi-centre [77]	2003–2009	CRT + surgery + GEM	33	17.4	Terminated—slow recruitment.
		Surgery + GEM	33	14.4 (*P* = 0.79)	
Bologna [78]	2007–2014	CRT + surgery	18	19.5	Terminated—slow recruitment.
		Surgery only	20	22.4 Not significant	
PACT-15 [79]	2010–2015	PEXG + surgery + PEXG	26	–	≤75 years, stage I–II, protocol event-free at 1 year: 6 (23%, 95% CI, 7–39) of 30; 15 (50%, 32–68) of 30; 19 (66%, 49–83) of 29.
		Surgery + GEM	30	–	
		Surgery + PEXG	32	–	
PREOPANC1 [80, 81]	2013–2017	CRT + GEM + Surgery + GEM	65	14.6	Longterm follow-up median OS HR 0.79 (95% CI, 0.54–1.16).
		Surgery + GEM	68	15.6 (*P* = 0.83)	
SWOG S1505 [82]	2015–2018	mFOLFIRINOX + Surgery + mFOLFIRINOX	55	23.2	Primary end point >2-year OS of 40%: 47 (95% CI, 31–61)% for arm 1 and 48 (31–63)% for arm 2.
		GEM-NabP + Surgery + GEM-NabP	47	23.6 Not significant	
NEONAX-AIO-PAK-0313 [83]	2015–2021	GEM-NabP + surgery + GEM-NabP	Resected 48/63	25.2	Planned 166 patients. Primary endpoint median DFS rate of 55% at 18 months double negative result: neoadjuvant 32.2%: adjuvant 41.4%.
		Surgery + GEM	Resected 51/64	16.7 Not significant	
PANACHE01-PRODIGE48 [84]	2017–2020	mFOLFIRINOX + Surgery + CTX	70	30.6	CTX = Initially GEM, 5FU and GEMCAP, but latterly and mostly mFOLFIRINOX.
		FOLFOX + Surgery + CTX	50	31.3	
		Surgery + CTX	26	36 Not significant	
NORPACT-1 [85]	2017–2021	mFOLFIRINOX + Surgery + mFOLFIRINOX	77	25.1	Primary endpoint was patients alive at 18 months: 60% in the neoadjuvant group versus 73% in the upfront surgery group (*p* = 0·032); upfront surgery had longer survival.
		Surgery + mFOLFIRINOX	63	38.5 (*P* = 0.050)	
PREOPANC2 [86]	2018–2021	FOLFIRINOX + Surgery	Resected 99/120	–	For all cases mixed resectable and borderline median survival = 21.9 months chemo vs 21.3 months CRT, *p* = 0.32.
		GEM + CRT + Surgery + GEM	Resected 95/121	–	
NEPAFOX [87]	2015–2018	Surgery + GEM	21	25.7	Mixed resectable (76%) and borderline resectable (79%); trial closed due to slow accrual target = 126 patients.
		mFOLFIRINOX + Surgery + mFOLFIRINOX	19	10.0	
Prep-02/JSAP-05 [88]	2013–2016	GEM + S1 +Surgery+ S1	131	HR = 0.73 (95% CI = 0.56, 0.95; *P* = 0.018)	Adjuvant CTX ‘advised ‘ but not required. ECOG = 0/1, <80 years. The study includes both resectable (*n* = 163) and borderline (*n* = 68), results are not cleanly separated.
		Surgery + S1	132		
Zhejiang [89]	2018–2024	GEM-NabP + mFOLFIRINOX+ Surgery+ Gem-CAP or mFOLFIRINOX	Resected 135/162	35.4 (95% CI = 27.9, 5.1)	Left pancreatectomies in 147 (52%); CA19-9 > 500 KU/L in 138 (43%); R0 in 237 (83%). CTX ‘recommended’ but not required; 169 (52%) ‘completed’ CTX. Short follow-up median = 18.7 months.
		Surgery+ Gem-CAP or mFOLFIRINOX	Resected 149/162	27.2 (95% CI = 19.8, 33.5) (*P* = 0.048)	

*CRT* chemoradiotherapy, *GEM* gemcitabine, *PEXG* cisplatin, epirubicin, gemcitabine and capecitabine, *CTX* chemotherapy, *5FU* 5-fluorouracil, *CAP* capecitabine, *mFOLFIRINOX*, modified folinic acid (FA), 5-fluorouracil (5FU), irinotecan (IR) and oxaliplatin (OX), *NabP* nab-paclitaxel, *DFS* disease free survival, *OS* overall survival.

As we know from the ESPAC and the PRODIGE studies, chemotherapy dose intensities are critical in determining overall survival, but this information is lacking in Prep-02/JSAP05 and both groups were ‘*strongly advised*’ to take adjuvant therapy, but not required to do so [15, 16, 88]. The overall survival results are worse than the JASPAC-01 trial presumably because of the inclusion of patients with EmBR tumours as well as the apparent lower total chemotherapy doses [8, 88]. The overall survival with upfront surgery and ‘advised’ adjuvant S-1 was 26.6 months (95% CI, 21.5–31.5) In JSAP-05 compared to 46·5 months (95% CI, 37·8–63·7) with upfront surgery and required adjuvant S-1 in JASPAC-01; the overall survival with neoadjuvant gemcitabine plus S-1, surgery and advised adjuvant S-1 in JSAP-05 was only 37.0 months (95% CI, 28.6–43.3) [8, 88]. As aforementioned this is where comparing the ‘resected’ patients of adjuvant trials with the ‘resectable’ patients of neo-adjuvant trials is problematic. The authors concluded that ‘The Prep-02/JSAP05 trial results showed that neoadjuvant chemotherapy with gemcitabine plus S-1 significantly extends survival compared with upfront surgery in patients with resectable PDAC’ but this statement does not seem to be supported by the data [88]. The Clinical Practice Guidelines for Pancreatic Cancer 2022 of the Japan Pancreas Society does not strongly recommend this approach [89, 90].

The 2 × 2 factorial CASSANDRA superiority trial was conducted in 260 patients with EmR/EmBR PDAC tumours aged 75 years or less [28]. In the first randomisation patients were enroled to either 4 months induction PAXG (capecitabine, cisplatin, nab-paclitaxel and gemcitabine) or to 4 months induction mFOLFIRINOX. Then in the second randomisation they were allocated to either 2 more months of chemotherapy before surgery and no further chemotherapy or surgery followed by 2 months adjuvant chemotherapy. The primary endpoint was event-free survival (EFS) at 3 years defined as one or more of the following: no progression, no recurrence, two consecutive CA19-9 increases by 20% separated by 4 or more weeks, non-resection, intraoperative metastasis and death. The preliminary findings of the first randomisation were presented at ASCO 2025, but for an unspecified reason not the second randomisation. After a median follow-up of 23.9 months, the 3 year median (95% CI) EFS with PAXG was 30% (20, 40) with 14% (5–23) with mFOLFIRINOX with an HR of 0.66 (0.49–0.89; *p* = 0.005) [28]. The trial however included patients with both EmR (*n* = 126) and EmBR (*n* = 134) cancers with 3 year median EFS rates according to the forest plot in the EmR patients of 47.2 vs 22.0% respectively and in the EmBR patients of 18.8 vs 8.9% respectively. The median overall survival in the mixed groups was 37.3 months (26.9-not reached) in the PAXG group and 26.0 months (23.6–39.4) in the mFOLFIRINOX group with an HR of 0.70 (0.47,1.04; *P* = 0.07) [28]. As well as showing no significant difference for overall survival between the two groups on preliminary analysis, dose intensities were not mentioned in the presentation [28]. The primary endpoint of event free survival as defined in this study does not seem to have been previously validated. There was no control comparator of standard therapy, namely immediate surgery plus adjuvant treatment for the EmR subgroup, and no data on the second randomisation of 2 more months additional chemotherapy prior to surgery versus surgery then 2 months adjuvant chemotherapy therefore indicating differences in, and potentially suboptimal, adjuvant chemotherapy.

The PREOPANC2 trial compared neoadjuvant full-dose FOLFIRINOX but with no scheduled adjuvant chemotherapy versus neoadjuvant chemoradiotherapy and gemcitabine plus adjuvant gemcitabine in both EmR and EmBR patients with no significant differences in overall survival [86]. In other words, there was no proper control group for the EmR patients which is resection followed by 6 months adjuvant mFOLFIRINOX. Survival estimates for separated resected EmR, EmBR, FOLFIRINOX and chemoradiotherapy groups were not provided preventing inter-trial comparisons.

The recent single centre study from Zhejiang University Hospital, Hangzhou, randomised 324 patients with EmR PDAC to neoadjuvant Gem-NabP plus mFOLFIRINOX or upfront surgery [89]. Patients in the neoadjuvant therapy group received nab-paclitaxel and gemcitabine concurrently on days 1, 8 and 15, followed by mFOLFIRINOX on days 29 and 43. Four cycles of adjuvant GemCap were ‘recommended’ in the neoadjuvant group and six cycles of adjuvant GemCap were ‘recommended’ for the upfront surgery group [89]. From 135 patients (83%) resected in the neoadjuvant group 112 patients started adjuvant therapy (69% of those randomised; 83% of those resected) of whom 84 patients (75%) had mFOLFIRINOX and 11 patients (10%) had GemCap. From 149 patients (92%) resected in the upfront surgery group 114 patients started adjuvant therapy (70% of those randomised; 76% of those resected) of whom 86 patients (75%) had mFOLFIRINOX and 10 patients (9%) had GemCap. Only 169 (52%) patients randomized ‘completed’ chemotherapy: 89 patients (55%) in the neoadjuvant group and 80 patients (49%) in the upfront surgery group and most importantly no total dose intensities for either group were provided. The median overall survival was 35.4 months (95% CI, 27.9–45.1) in the neoadjuvant therapy group versus 27.2 months (95% CI, 19.8–33.5) in the upfront surgery group (HR, 0.73; 95% CI, 0.53–1.00; *p* = 0.0477) [89]. As well as being potentially biased as an open labelled single centre trial and unspecified adjuvant therapy, the conclusions of this trial are further questionable by the short median follow-up (18.7 months, interquartile range 13.0, 32.0), a high proportion of patients with a CA19-9 > 500KU/L (in 138, 43%), an unusually low R0 rate (in 237, 83%) and a very high proportion of left pancreatectomies (in 147, 52%) [89].

Of particular interest is the NORPACT-1 multicenter trial from hospitals in Denmark, Finland, Norway and Sweden, that randomized patients with resectable PDAC to receive either neoadjuvant mFOLFIRINOX (four cycles before surgery then adjuvant chemotherapy) or upfront surgery group (followed by adjuvant chemotherapy) [85]. Initially, adjuvant chemotherapy was GemCap (four cycles in the neoadjuvant group, and six cycles in the upfront surgery group), and was subsequently amended to permit use of adjuvant mFOLFIRINOX (eight cycles in the neoadjuvant group, and 12 cycles in the upfront surgery group) [85]. The primary endpoint was overall survival at 18 months. The proportion of patients alive at 18 months was 60% (95% CI. 49–71) in the 77 patients in the neoadjuvant group versus 73% (95% CI, 62–84) in the 63 patients in upfront surgery group (*p* = 0·032). The median overall survival was 25·1 months (95% CI, 17·2–34·9) versus 38·5 months (95% CI, 27·6–not reached) respectively, with a hazard ratio of 1·52 (95% CI, 1·00–2·33; *p* = 0·050). ^85^ Thus not only was the primary endpoint not reached but patients who underwent upfront surgery with adjuvant chemotherapy had longer survival [85]. Even though it has not been shown beneficial to use a neoadjuvant approach for the EmR group as a whole this does not necessarily rule out that certain biological subgroups of patients—yet to be established—would benefit from a neoadjuvant approach, which future studies should try to address.

## Advantages and disadvantages of adjuvant therapy with mFOLFIRINOX versus gemcitabine-capecitabine

The long-term follow-up results from both ESPAC4 and PRODIGE 24 have enabled some important information of how these regimens can be used optimally for particular patients [16, 18].

Long-term follow up of 732 patients randomized in the ESPAC4 trial confirmed the overall and relapse-free survival superiority of the gemcitabine-capecitabine combination over gemcitabine. The GemCap combination produced particularly striking survival results in patients with resection margin free (R0) tumours and lymph node negative resections [18]. The gemcitabine-capecitabine combination in R0 patients had a median overall survival of 49.9 months (95% CI, 39.0–82.3) compared to 32.2 months (95% CI, 27.9–41.6) with gemcitabine and a 5 year overall survival of 45% (95% CI, 38–54) versus 31% (95% CI, 25–40) respectively with a hazard ratio of 0.63 (0.47–0.84; *p* = 0.002) [16]. Interestingly the PRODIGE24 trial found an *opposite effect* with a non-significant hazard ratio of 0.86 (95% CI, 0.63–1.19) in patients with R0 tumours for mFOLFIRINOX vs gemcitabine but with a significant effect in patients with R1 tumours with a hazard ratio of 0.60 (95% CI, 0.43–0.83) [16].

In ESPAC4 patients with lymph node negative tumours had significantly higher 5 year overall survival rates with gemcitabine-capecitabine of 59% (95% CI, 49–71) than gemcitabine showing 53% (95% CI, 42–66), with a hazard ratio of 0.63 (95% CI, 0.41 – 0.98) (*p* = 0.04) but not those with positive lymph nodes (*p* = 0.225) [18]. Again, the *opposite effect* was seen in PRODIGE24 with a non-significant hazard ratio of 1.07 (95% CI, 0.63–1.81) for mFOLFIRINOX vs gemcitabine in patients with lymph node negative tumours compared to a significant hazard ratio of 0.63 (95% CI, 0.49–0.81) in patients with lymph node positive tumours [16]. In a sense these findings might be considered to mirror the contrasting effects between these two regimens in advanced disease, with FOLFIRINOX being more effective than gemcitabine in the metastatic setting but not in locally advanced disease whilst gemcitabine-capecitabine seems more effective than gemcitabine in patients with locally advanced disease but less so for metastatic [16, 18, 91–94]. Note that in the NEOPAN study which randomized patients with locally advanced pancreatic cancer to FOLFIRINOX or gemcitabine monotherapy, the median progression free survival (the primary end-point) was 9.7 months (95% CI, 7.0–11.7) versus 7.7 months (95% CI, 6.2–9.2) respectively, with a hazard ratio of 0.7 (95% CI, 0.5–1.0; *p* = 0.04) [94]. On the other hand the median overall survival was 15.7 months (95% CI, 11.9–20.4) in the FOLFIRINOX group compared to 15.4 months (95% CI, 11.7–18.6) in the gemcitabine group with a hazard ratio of 1.02 (95% CI, 0.73–1.43; *p* = 0.95), somewhat reflecting the findings of the ESPAC5 neoadjuvant randomized trial with not dissimilar overall survival between the GemCap and mFOLFIRINOX groups [94, 95].

In the long term follow-up study of ESPAC4, it was found that the median relapse-free survival rate was 18.3 months (95% CI, 16.3–21.0) in the gemcitabine group and 21.3 months (95% CI, 18.3–24.5) in the gemcitabine-capecitabine group with a hazard ratio of 0.85 (95% CI, 0.72–1.00; *p* = 0.053) [18]. These results are now very similar to the PRODIGE24 findings with a median disease-free survival of 12.8 months (95%CI, 11.6–15.2) with gemcitabine and 21.4 months (95%CI, 17.5–26.7) with mFOLFIRINOX with a hazard ratio of 0.66 (95%CI, 0.54–0.82; *p* < 0.001) [16]. It is not clear why the median disease-free survival rates are apparently similar whilst the overall survival rates are disparate, but may relate to greater use of second line therapies in PRODIGE24, and or different biological effects between GemCap and mFOLFIRINOX.

The ESPAC4 long term follow-up study also found significantly improved overall and relapse-free survival for gemcitabine-capecitabine over gemcitabine in the 193 (26.4%) of 732 ESPAC4 patients who would not have been eligible for mFOLFIRINOX using the PRODIGE24 trial criteria [18]. This is a very important finding for standard clinical practice showing that if a patient is not eligible for mFOLFIRINOX then they should be offered gemcitabine-capecitabine rather than gemcitabine since the manageable toxicities of the latter two are comparable yet the overall survival benefits are greater with the gemcitabine-capecitabine combination.

## Precision targeting of chemotherapy

Due to tumour heterogeneity and plasticity, it has become apparent that PDAC tumours will respond non-homogeneously to one or other cytotoxic drug regimen [68, 73–75]. A variety of transcriptomic signatures have been developed to predict responses to gemcitabine-based versus oxaliplatin-based regimens based on the initial work by David Tuveson and Steve Gallinger that was focused on human derived pancreatic cancer organoids [96]. The PurIST (Purity Independent Subtyping of Tumours) signature is a 16-gene single sample classifier that can distinguish patients more likely to respond to mFOLFIRINOX rather than gemcitabine plus nab-paclitaxel [97–99]. Initial studies have suggested that in advanced PDAC patients with PurIST classical-like tumours and low ECOG scores have longer overall survival with first line mFOLFIRINOX treatment compared to first line gemcitabine plus nab-paclitaxel [99]. An open-label, phase II study in patients with resectable and borderline resectable pancreatic cancer using PurIST subtyping is allocating patients with classical-like to mFOLFIRINOX and patients with basal-like subtypes to gemcitabine plus nab-paclitaxel (ClinicalTrials.gov ID NCT04683315).

The GemPred RNA signature containing thousands of transcripts was derived from preclinical models, but predictions suffered from being associated with the basal-like and classical-like PDAC subtypes [100]. In an attempt to overcome this bias, patient derived pancreatic organoid sequencing data were included in the signature identification strategy, resulting in an ‘Improved GemPred’ signature [100]. This was further refined into the ‘GemCore’ signature containing < 100 transcripts and most recently into an artificial intelligence-driven tool called ‘PancreasView’ incorporating human PDAC stromal sequencing data [101, 102]. A post-hoc analysis of patients in the PRODIGE-24/CCTG PA6 trial showed that the ‘PancreasView’ Instrument could predict 30% of patients receiving gemcitabine monotherapy with comparable survival to those given mFOLFIRINOX, and with fewer adverse events [103]. The gemcitabine response prediction by PancreasView is independent of molecular subtype prediction but not the mFOLFIRINOX prediction [103]. Ongoing prospective studies are aimed at validation (NCT05475366, advanced PDAC, PACsign; NCT06046794, GemCore+ in metastatic PDAC, GemSign-01; NCT06046794, neoadjuvant for EmBR, NeoPREDICT). The GemciTest is a blood-based RNA signature, that measures the expression levels of nine genes using real-time polymerase chain reaction processes, which predicts longer survival in gemcitabine treated patients but not in fluoropyrimidine-based treated patients [104].

The NeoPancOne phase II study of peri-operative mFOLFIRINOX in patients with EmR PDAC, was recently reported at ASCO 2025 [105]. The aim of NeoPancOne is to prospectively investigate the expression levels of the transcription factor GATA6 which can distinguish between classical-like (high GATA6 expression) and basal-like (low GATA6 expression) molecular subtypes [106, 107]. The NeoPancOne study found that disease progression within 6 months occurred in nearly 50% of patients with low tumour GATA6 expression such that neoadjuvant mFOLFIRINOX might no longer be considered the standard of care in these patients [105]. The ESPAC6 randomized European multicenter trial aims to compare standard adjuvant therapy in EmR patients with an experimental arm in which patients are allocated to mFOLFIRINOX or gemcitabine plus capecitabine based on a 236 gene transcriptomic signature (ClinicalTrials.gov: NCT05314998). The ESPAC signature is unique in having been developed using machine learning exclusively from RNA sequencing of human PDAC tumours.

Precision targeting of chemotherapies is currently and in the foreseeable future very important, since while there is optimism for precision therapy using novel agents, there is still much caution required. Although targeted therapies for PDAC are applicable to perhaps less than 5% of patients at present, remarkable advances are now being made in targeted therapies and immunotherapies, but these novel treatments have yet to show a significant improvement in overall survival [69, 108]. In metastatic pancreatic cancer, despite FDA approval of first line NALIRIFOX (nanoliposomal irinotecan, 5FU and oxaliplatin) based on the NAPOLI trial and olaparib maintenance for patients with germline BRCA variants based on the POLO trial, both trials have been these criticised as producing positive results due to schemed trial design rather than being aimed at improving patient outcomes [109–112]. It has been strongly argued that the principles governing Common Sense Oncology should form the basis for improving the design, analysis and reporting of randomized controlled phase III clinical trials evaluating cancer treatments, emphasising that control treatment should be the best current standard of care and that the preferred primary endpoint is overall survival or a validated surrogate [113].

## Conclusions: main findings and implications

Current practice and future trends are itemised in the Summary Box 1. The NRG Oncology/RTOG 0848 trial from the USA of adjuvant chemotherapy with or without chemoradiation, has shown that the addition of chemoradiation to systemic chemotherapy had no overall survival advantage compared to chemotherapy alone. In particular there was no survival benefit for patients with resection margin positive or lymph node positive tumours.

No randomized trial has shown convincingly that neoadjuvant therapy improves overall survival in patients with resectable as opposed to improved survival in patients with borderline resectable pancreatic cancer. The NORPACT-1 trial of neoadjuvant FOLFIRINOX versus upfront surgery from the Nordic countries, even showed that there was increased survival in patients that had upfront surgery with adjuvant therapy rather than patients that had neoadjuvant therapy.

Long-term follow-up of the France-Canada PRODIGE24 and the European ESPAC4 trials has confirmed mFOLFIRINOX as the reference standard for adjuvant chemotherapy. We learn however from the ESPAC4 trial that an additional 26% of patients can be treated with gemcitabine-capecitabine (rather than gemcitabine) who are not eligible (or decline) mFOLFIRINOX. Although there was no direct head-to-head comparison mFOLFIRINOX seems to especially benefit patients with resection margin positive or lymph positive tumours, whilst gemcitabine-capecitabine especially benefits patients with resection margin negative or lymph node negative tumours. In the near future molecular signatures may help to further refine selection of induction, neoadjuvant and adjuvant systemic therapies.

Box 1 Current practice and future trends for the treatment of resectable and localised pancreatic cancer The ESPAC1 and ESPAC1-Plus trials in 2001-2004 established surgery and 6 months adjuvant chemotherapy (but without chemoradiotherapy) as the standard of care.

Five year overall survival rates based on this paradigm have improved from less than 10% to 30–50%.

Without surgery there are no 5 year survivors.

Following ESPAC1 there was a rapid increase in the number of resections undertaken.

Resection with adjuvant chemotherapy is the single most contributing factor accounting for the increase in overall 5 year survival rates from <5% to 13% for all stages combined.

Empirical criteria have evolved to enhance clinical decision making, reflecting the discovery of clonal evolution and biotyping of PDAC tumours. EmR: resectable; EmBR: borderline resectable; EmUR: locally advanced, unresectable; EmOM: oligometastatic; EmPM: polymetastatic.

EmR: Six months mFOLFIRINOX or if not feasible GemCap following resection. No role for chemoradiotherapy.

EmBR: Short course neoadjuvant combination chemotherapy before surgery; role of chemoradiotherapy not proven.

EmUR: 4–6 months induction combination chemotherapy before surgery; role of chemoradiotherapy not proven.

Initial sampling and serial liquid biopsies for evolving tumour biotyping and minimal residual disease detection will be of increasing importance.

New perioperative treatment paradigms, including transcriptomic signature-guided chemotherapy and targeted therapy are being tested in clinical trials and will likely change the treatment landscape in the near future.
